# Hemodynamic effects of supplemental oxygen versus air in simulated blood loss in healthy volunteers: a randomized, controlled, double-blind, crossover trial

**DOI:** 10.1186/s40635-023-00561-z

**Published:** 2023-11-10

**Authors:** Sole Lindvåg Lie, Jonny Hisdal, Marius Rehn, Lars Øivind Høiseth

**Affiliations:** 1 Department of Research and Development, Norwegian Air Ambulance Foundation, Oslo, Norway; 2 Institute of Clinical Medicine, University of Oslo, Oslo, Norway; 3 Section of Vascular Investigations, Oslo University Hospital, Oslo, Norway; 4 Air Ambulance Department, Division of Prehospital Services, Oslo University Hospital, Oslo, Norway; 5 Department of Anesthesia and Intensive Care Medicine, Division of Emergencies and Critical Care, Oslo University Hospital, Oslo, Norway

**Keywords:** Cardiac output, Cerebrovascular circulation, Hemodynamic, Hypovolemia, Lower body negative pressure, Oxygen inhalation therapy, Randomized controlled trial, Trauma

## Abstract

**Background:**

Trauma patients frequently receive supplemental oxygen, but its hemodynamic effects in blood loss are poorly understood. We studied the effects of oxygen on the hemodynamic response and tolerance to simulated blood loss in healthy volunteers.

**Methods:**

Fifteen healthy volunteers were exposed to simulated blood loss by lower body negative pressure (LBNP) on two separate visits at least 24 h apart. They were randomized to inhale 100% oxygen or medical air on visit 1, while inhaling the other on visit 2. To simulate progressive blood loss LBNP was increased every 3 min in levels of 10 mmHg from 0 to 80 mmHg or until hemodynamic decompensation. Oxygen and air were delivered on a reservoired face mask at 15 L/min. The effect of oxygen compared to air on the changes in cardiac output, stroke volume and middle cerebral artery blood velocity (MCAV) was examined with mixed regression to account for repeated measurements within subjects. The effect of oxygen compared to air on the tolerance to blood loss was measured as the time to hemodynamic decompensation in a shared frailty model. Cardiac output was the primary outcome variable.

**Results:**

Oxygen had no statistically significant effect on the changes in cardiac output (0.031 L/min/LBNP level, 95% confidence interval (CI): − 0.015 to 0.077, P = 0.188), stroke volume (0.39 mL/LBNP level, 95% CI: − 0.39 to 1.2, P = 0.383), or MCAV (0.25 cm/s/LBNP level, 95% CI: − 0.11 to 0.61, P = 0.176). Four subjects exhibited hemodynamic decompensation when inhaling oxygen compared to 10 when inhaling air (proportional hazard ratio 0.24, 95% CI: 0.065 to 0.85, P = 0.027).

**Conclusions:**

We found no effect of oxygen compared to air on the changes in cardiac output, stroke volume or MCAV during simulated blood loss in healthy volunteers. However, oxygen had a favorable effect on the tolerance to simulated blood loss with fewer hemodynamic decompensations. Our findings suggest that supplemental oxygen does not adversely affect the hemodynamic response to simulated blood loss.

*Trial registration* This trial was registered in ClinicalTrials.gov (NCT05150418) December 9, 2021

**Supplementary Information:**

The online version contains supplementary material available at 10.1186/s40635-023-00561-z.

## Introduction

Supplemental oxygen is frequently administered to trauma patients to prevent arterial hypoxemia and tissue hypoxia [1, 2]. However, the evidence supporting this is limited [3], and high doses may lead to hyperoxia, associated with poor clinical outcomes in mixed patient populations [4]. Studies involving healthy volunteers have demonstrated that hyperoxia reduces cardiac stroke volume, heart rate, and cardiac output [5], as well as perfusion of the brain [6]. In traumatic blood loss, venous return is decreased resulting in diminished stroke volume initiating a compensatory rise in heart rate. However, this response is insufficient to sustain cardiac output. If oxygen has similar effects in blood loss as in normovolemic conditions, the resulting cardiac output might be critically low. As blood loss is the leading cause of preventable deaths in trauma patients [7, 8] and supplemental oxygen is recommended in guidelines [9–11], understanding its hemodynamic effects is crucial.

Lower body negative pressure (LBNP) is a validated model of blood loss in healthy volunteers [12]. One previous study investigated the effect of supplemental oxygen during LBNP, but was unblinded and used only a single, moderate LBNP level of short duration which makes it unclear whether oxygen influences the systemic hemodynamic response to blood loss [13].

The aim of the present trial was therefore to study the effect of supplemental oxygen compared to air on the hemodynamic response to simulated blood loss. We hypothesized that the changes in cardiac output during simulated blood loss would be different when inhaling oxygen compared to air.

## Methods

### Trial design

This study was a single-center, experimental, randomized, controlled, double-blind, crossover trial. A crossover design was chosen since within-subject variation is less than between-subject variation, reducing the required number of participants. Subjects participated on two visits at least 24 h apart. Visits 1 and 2 were on a similar time of day.

### Subjects

Healthy subjects between 18 and 50 years of age were recruited according to the eligibility criteria provided in Additional file 1. Exclusion criteria included any condition limiting physical exertional capacity, regular medication use (except allergy and contraceptives), pregnancy, breastfeeding, history of syncope (excluding presumed vasovagal) and cardiac arrhythmia. All subjects provided a written informed consent. They were allowed to have a light meal on the day of the experiment, having as a minimum abstained from caffeine (6 h), nicotine (12 h) and strenuous exercise (3 h) prior to the visit.

### Interventions

100% oxygen or medical air (21% oxygen) was delivered on a reservoired face mask at 15 L/min and is referred to as “treatment”. Except for the treatment, the visits were identical.

**Fig. 1:**
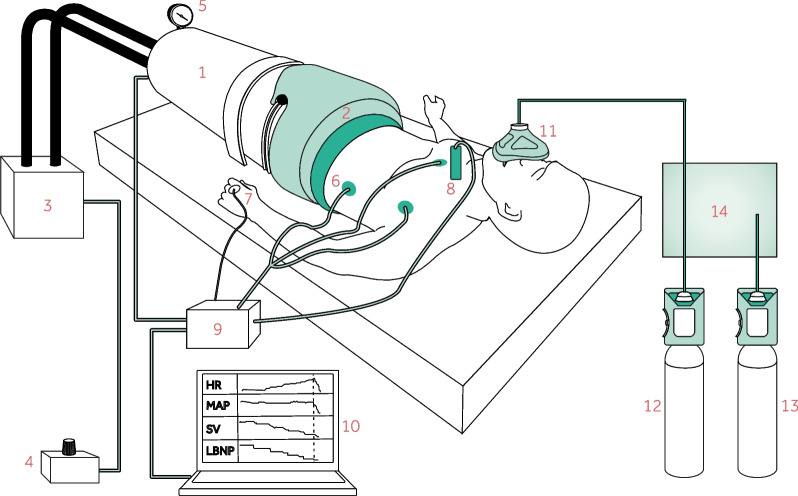
Lower body negative pressure (LBNP) model, graphics reused from protocol under CC BY 4.0 license [14]. The subject was positioned inside (1) the LBNP chamber which was (2) sealed just above the level of the iliac crest and connected to (3) a vacuum pump controlled by (4) a pressure control unit. The chamber pressure was displayed on (5) a pressure monitor. Hemodynamic variables including (6) heart rate (HR) from an ECG, (7) mean arterial pressure (MAP) from a finger cuff and (8) stroke volume (SV) from Doppler ultrasound were transmitted to (9) a data acquisition device and (10) sampled on a computer continuously. The treatment was administered on (11) a reservoired face mask. (12, 13) Two cylinders containing oxygen and air were connected to (14) a concealment container from which only one tube continued to the face mask. Whether the tube connected to the oxygen or air cylinder continued to the subject was given by the randomization list

**Fig. 2:**
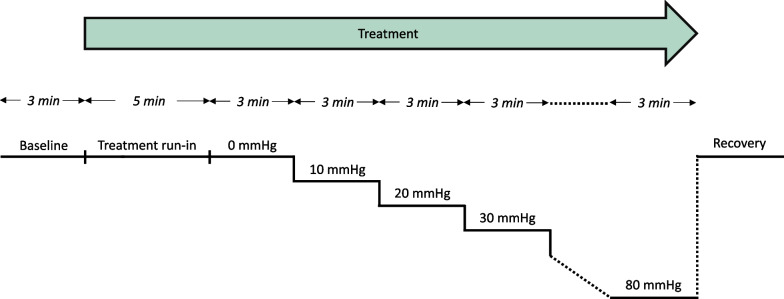
Lower body negative pressure (LBNP) protocol. After a 3-min baseline period and 5-min run-in time of the treatment (oxygen or air), LBNP was increased by 10 mmHg starting from 0 mmHg every 3 min to simulate progressive blood loss until completing LBNP 80 or stopping due to hemodynamic decompensation

Prior to the experiments, the subjects underwent familiarization with the experimental setup. Room temperature was kept between 22 and 24 degrees Celsius. At each visit, the following standardized protocol was implemented. The subjects were positioned in the LBNP chamber, as shown in Fig. 1, and remained supine for 20–30 min to allow hemodynamic variables to stabilize. Subsequently, a 3-min baseline period was recorded during which the subjects inhaled ambient room air without a face mask. Thereafter, the subjects were instructed to inhale the treatment through a face mask for the entire LBNP protocol, starting with a 5-min run-in period. LBNP was then increased stepwise in levels of 10 mmHg every 3 min, starting from LBNP 0 (no blood loss), until completing LBNP 80 mmHg (as illustrated in Fig. 2) or until the experiment was stopped due to hemodynamic decompensation given by the following criteria: symptoms of pre-syncope (light-headedness, nausea or sweating), reductions in MAP or heart rate to less than 75% of baseline values for > 3 s or subject request for reasons other than above.

### Randomization and blinding

Subjects were randomly assigned in a 1:1 ratio to inhale either oxygen or air on visit 1, and the opposite on visit 2. Block randomization with block sizes of 4 or 6 was used to attain a balanced design, utilizing the “blockrand” package [15] in R [16]/Rstudio [17]. A randomization list was automatically created and handed to a third party who prepared inhalation gas tubing corresponding to the intended treatment. Blinding and allocation concealment were ensured using a non-translucent concealment container (Fig. 1) through which one gas tube was connected to the face mask while the other remained blind inside the container.

### Trial oversight

The trial was approved by the Regional Ethics Committee (REK South East D, ref. 285164) and the Norwegian Medical Agency (21/15284-9) and registered in ClinicalTrials.gov (NCT05150418) and in the European Union Drug Regulating Authorities Clinical Trials Database (2021-003238-35). The protocol was published in advance [14]. The Clinical Trials Unit at Oslo University Hospital monitored the trial. This report follows CONSORT guidelines [18] (reporting checklist in Additional file 2).

### Measurements and data processing

Heart rate was obtained with a three-lead ECG (Bio Amp/PowerLab, ADInstruments, Bella Vista, Australia). Aortic blood velocity was measured continuously by suprasternal pulsed Doppler ultrasound (SD-50; Vingmed Ultrasound, Horten, Norway). Stroke volume was calculated as the product of aortic blood velocity–time integral and left ventricular outflow tract (LVOT) area from echocardiographic measurements obtained in parasternal long-axis [19]. Cardiac output was calculated as the product of heart rate and stroke volume. MAP was measured non-invasively via the volume-clamp method on the third finger of the left hand (Nexfin; BMEYE, Amsterdam, The Netherlands). Systemic vascular resistance (SVR) was calculated as the quotient of MAP and cardiac output. Peripheral oxygen saturation (SpO_2_) was measured by finger pulse oximetry (Masimo Radical 7; Masimo corp., CA, USA). Middle cerebral artery blood velocity (MCAV) was measured using triplex transcranial ultrasound (Vivid E95; GE Vingmed, Horten, Norway) as described elsewhere [20]. The time averaged maximum velocity for MCAV was measured automatically beat-to-beat in EchoPAC 202 (GE Vingmed, Horten, Norway). Cerebral tissue oxygen saturation (ScO_2_) was measured by near-infrared spectroscopy (NIRS, Invos 5100C cerebral/somatic oximeter; Somanetics, Troy, MI, USA). Sensors were placed on the subject’s left and right forehead and sampled every 7–8 s. Tolerance to simulated blood loss was measured as the time from the beginning of LBNP 0 until completing LBNP 80 or hemodynamic decompensation.

Cardiac output, stroke volume, heart rate, MAP, SVR and SpO_2_ were sampled in Lab Chart 8.1.9 (ADInstruments, Bella Vista, Australia) at 1000 Hz, and downsampled to beat-to-beat values. For data analyses, we calculated one mean value per LBNP level after removing the first minute of each LBNP level (to allow for hemodynamic stabilization) and trimming the 5% highest and lowest values (to remove noise caused by extrasystoles, movement artifacts, etc. in an objective and reproducible manner). All variables were visually inspected for errors. This constituted the dataset used in the analyses (Additional file 3).

### Outcomes

The primary outcome was the effect of oxygen compared to air on changes in cardiac output during simulated blood loss by LBNP. There were three secondary outcomes. The effect of oxygen compared to air on changes in (1) stroke volume and (2) MCAV during simulated blood loss, and (3) the effect on tolerance to simulated blood loss.

In addition to the prespecified outcomes, we also estimated the effect of oxygen on heart rate, MAP, SVR and ScO_2_.

### Statistics

Based on a previous study [21], and that a 15% change in cardiac output often is considered clinically significant [22], simulations showed that 15 subjects would detect a 15% reduction in cardiac output with oxygen compared to air with 1 − β = 0.87 and α = 0.05. We assumed a mean cardiac output of 4.85 ± 1.08 L/min at baseline, a 0.245 L/min reduction per 10 mmHg of LBNP and a within-subject standard deviation (SD) of 0.385 L/min [14].

To account for repeated measurements within subjects, the effect of oxygen compared to air on the absolute changes in the outcome variables during LBNP was analyzed with mixed linear regression with subjects as a random effect using the “nlme” package in R [23]. The outcome variable was entered as the response variable while LBNP level and treatment, including their interaction effects, were entered as explanatory variables. The interaction term of LBNP level and treatment was considered the treatment specific effect on the response variable. The main effect of treatment in the regression model was considered the effect from baseline to LBNP. We compared this parsimonious model to models including polynomial terms of LBNP level up to power two, including an interaction with treatment, to account for a potential non-linear change in the response variable with LBNP using Akaike information criterion. LBNP level was treated as a continuous variable. Treatment was entered as a factor.

Tolerance to simulated blood loss was analyzed using shared frailty models with the event being hemodynamic decompensation and survival time being time to hemodynamic decompensation and subject as a random effect using the “survival” package in R [24]. Censoring occurred when completing LBNP 80. Probability of hemodynamic stability was defined as 1 − proportion of subjects exhibiting hemodynamic decompensation.

Precisions in MCAV and stroke volume measurements were calculated on baseline data at rest without treatment. The baseline period of 3 min was divided into 1-min averages and analyzed in a mixed linear regression model with subjects, and visits nested within subjects, as a random intercept. Precision was calculated as 1.96 × SD of residuals.

P-values < 0.05 were considered statistically significant. Data are presented as mean (± SD). Model assumptions were checked by plotting standardized residuals vs. fitted values, QQ-plots and histograms of the residuals. Regression outputs are provided in Additional file 4 and R-code with statistical analyses in Additional file 5.

## Results

**Fig. 3:**
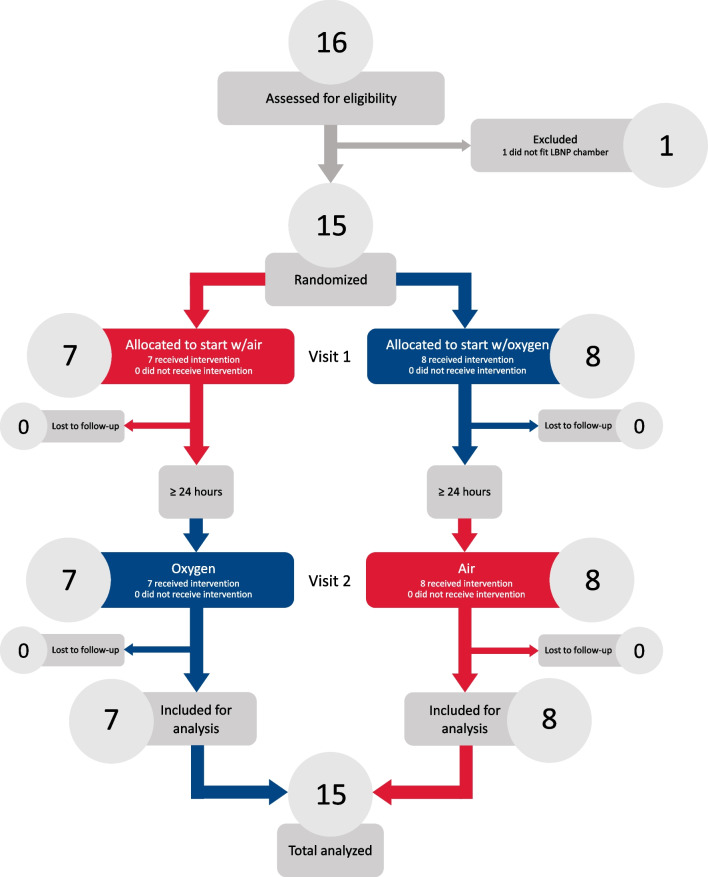
CONSORT subject flow diagram. Except for one subject who failed to fit inside the lower body negative pressure (LBNP) chamber all subjects completed both visits

From December 2021 to June 2022, we screened 16 and enrolled 15 subjects (7 female) with age 28 (7) years, height 176 (8) cm, weight 73 (13) kg and body mass index 23 (3) kg/m^2^ (Fig. 3). There was a separation in SpO_2_ between oxygen and air visits (Additional file 4, Figure S1).

### Primary outcome—cardiac output

**Fig. 4:**
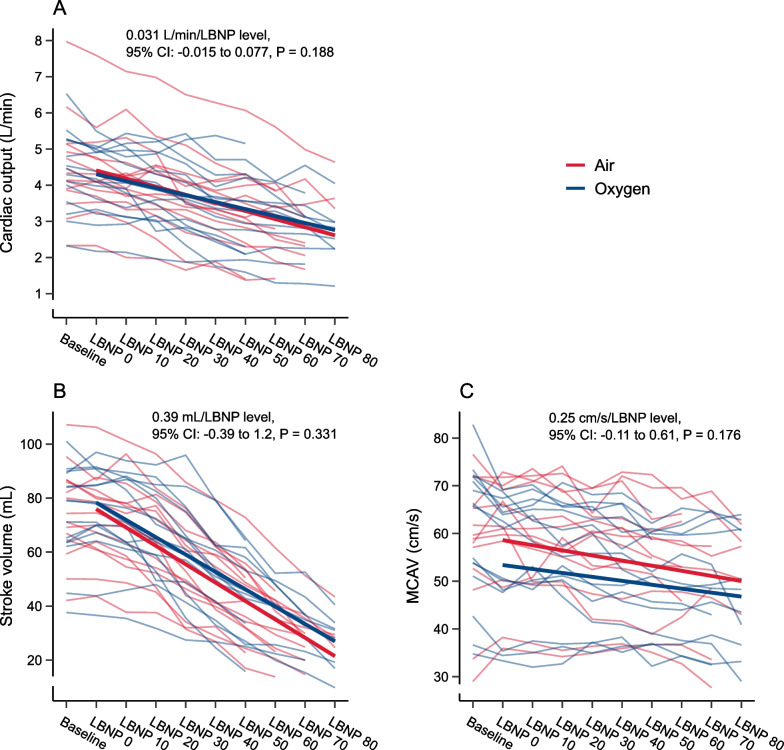
Cardiac output (**A**), stroke volume (**B**) and middle cerebral artery blood velocity (MCAV, **C**) during lower body negative pressure (LBNP) from 0 to 80 mmHg with oxygen (blue) and air (red). Thin lines represent subjects. Thick lines are regression estimates of the effect of oxygen and air on the changes in the outcome variable during LBNP

We found no difference in the changes in cardiac output during LBNP with oxygen compared to air (0.031 L/min/LBNP level, 95% confidence interval (CI): − 0.015 to 0.077, P = 0.188, Fig. 4A). There was no statistically significant main effect of oxygen on cardiac output from baseline to LBNP (− 0.10 L/min, 95% CI: − 0.30 to 0.096, P = 0.312).

### Secondary outcomes

#### Stroke volume

The precision in stroke volume measurements was ± 5.5 mL. We found no difference in the changes in stroke volume during LBNP with oxygen compared to air (0.39 mL/LBNP level, 95% CI: − 0.39 to 1.2, P = 0.331, Fig. 4B). There was no statistically significant main effect of oxygen on stroke volume from baseline to LBNP (2.4 mL, 95% CI: − 0.92 to 5.8, P = 0.154).

### Middle cerebral artery blood velocity

The precision in MCAV measurements was ± 4.1 cm/s. We found no difference in the changes in MCAV during LBNP with oxygen compared to air (0.25 cm/s/LBNP level, 95% CI: − 0.11 to 0.61, P = 0.176, Fig. 4C). There was a statistically significant main effect of oxygen on MCAV, corresponding to lower values during LBNP (− 5.2 cm/s, 95% CI: − 6.8 to − 3.7, P < 0.001).

### Tolerance to simulated blood loss

**Fig. 5:**
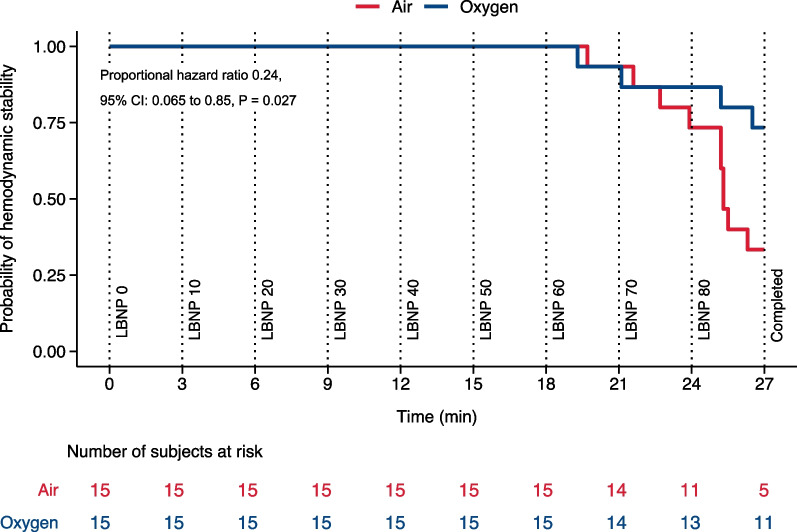
Kaplan–Meier estimates of probability of hemodynamic stability with oxygen (blue) and air (red) representing tolerance to simulated blood loss. “Time” is the duration of the visit in minutes. Vertical lines show where LBNP was increased and LBNP 80 completed

Four out of 15 subjects exhibited hemodynamic decompensation during the oxygen visits, versus 10 out of 15 subjects during the air visits. Three out of four decompensations during oxygen visits and 8 out of 10 decompensations during air visits were due to MAP decrease. The risk of hemodynamic decompensation decreased with oxygen compared to air (proportional hazard ratio 0.24, 95% CI 0.065 to 0.85, P = 0.027, Fig. 5).

### Explorative analyses

**Fig. 6:**
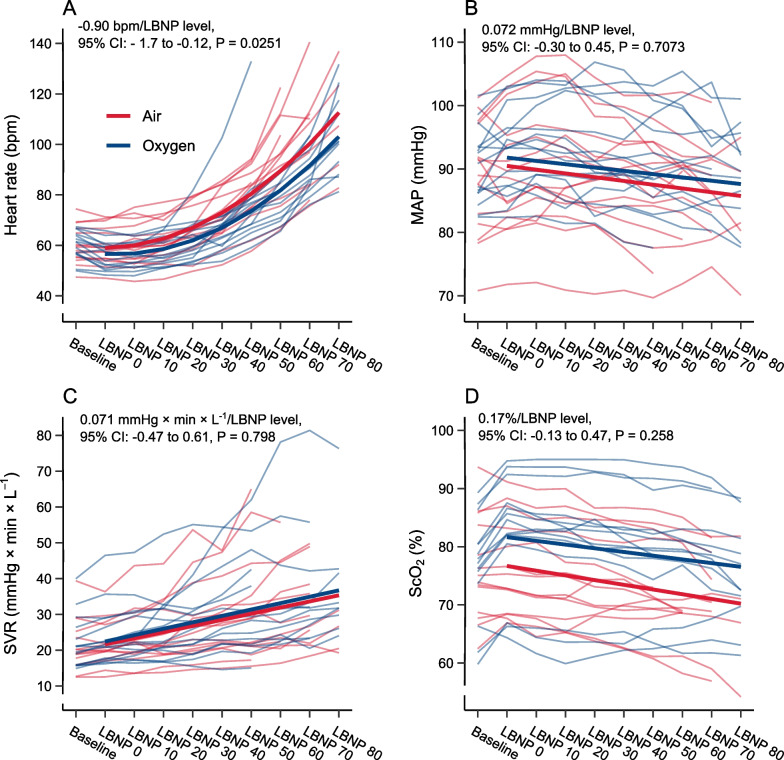
Heart rate (**A**), mean arterial pressure (MAP, **B**), systemic vascular resistance (SVR, **C**) and cerebral tissue oxygen saturation (ScO_2_, **D**) during lower body negative pressure (LBNP) from 0 to 80 mmHg with oxygen (blue) and air (red). Thin lines represent subjects. Thick lines are regression estimates of the effect of oxygen and air on the changes in the outcome variable during LBNP

There was a small but statistically significant difference in the changes in heart rate during LBNP with oxygen compared to air (− 0.90 bpm/LBNP level, 95% CI: − 1.7 to − 0.12, P = 0.0251, Fig. 6A). We found no difference in the changes in MAP (0.072 mmHg/LBNP level, 95% CI: − 0.30 to 0.45, P = 0.7073, Fig. 6B), SVR (0.071 mmHg × min × L^−1^/LBNP level, 95% CI: − 0.47 to 0.61, P = 0.798, Fig. 6C) or ScO_2_ (0.17%/LBNP level, 95% CI: − 0.13 to 0.47, P = 0.258, Fig. 6D) during LBNP with oxygen compared to air. There was a statistically significant main effect of oxygen on ScO_2_, corresponding to higher values during LBNP (5.0%, 95% CI: 3.6 to 6.3, P < 0.001). There were no statistically significant main effects of oxygen on heart rate (− 2.3 bpm, 95% CI: − 5.8 to 1.1, P = 0.183), MAP (1.3 mmHg, 95% CI: − 0.35 to 3.0, P = 0.121) or SVR (0.81 mmHg × min × L^−1^, 95% CI: − 1.5 to 3.1, P = 0.497) from baseline to LBNP.

## Discussion

This single-center, experimental, randomized, controlled, double-blind, crossover trial did not demonstrate an effect of oxygen compared to air on the changes in the primary outcome cardiac output during simulated blood loss in healthy volunteers. Neither did we find an effect of oxygen on the changes in the secondary outcomes stroke volume or MCAV. However, we observed a reduction in MCAV occurring before LBNP exposure that persisted during LBNP. Interestingly, we did find that oxygen improved tolerance to simulated blood loss. In our exploratory analyses, we found an increase in ScO_2_ occurring before LBNP exposure that persisted during LBNP, and a small but statistically significant effect of oxygen on the changes in heart rate, yielding a less pronounced heart rate increase during LBNP.

Our findings suggest that supplemental oxygen does not affect the changes in cardiac output during simulated blood loss in healthy volunteers, which is consistent with the previous work [13]. Since we applied a greater range of LBNP than the previous study, our results also suggest that the magnitude of LBNP does not influence the effect of oxygen on cardiac output.

While few have explored how oxygen influences hemodynamics during LBNP, many studies have examined the effect of oxygen at rest. We did not find a statistically significant main effect of oxygen on cardiac output, which seemingly contrasts a meta-analysis reporting a mean reduction of 10.2% in cardiac output when inhaling 100% oxygen [5]. However, it is important to note that data used in our regression analysis included all LBNP levels, as our trial was not designed and powered to study the effect of oxygen without LBNP. Also, in contrast to most studies in this meta-analysis, the present trial had a randomized, double-blinded, crossover design.

It may seem paradoxical that we observed an effect of oxygen on heart rate but not on stroke volume and cardiac output, given that cardiac output is the product of the former two. While the effect of oxygen on stroke volume was not statistically significant, our results indicate a tendency toward an increase with oxygen.

From baseline to LBNP, we observed a reduction in MCAV with oxygen that persisted during, and was not altered by, LBNP. Despite contention, MCAV measured by transcranial Doppler ultrasound is assumed to reflect cerebral blood flow under certain conditions [25, 26]. Assuming a constant diameter of the middle cerebral artery, the present finding suggests that cerebral blood flow may be reduced by supplemental oxygen during simulated blood loss, but we observed no synergistic effect between oxygen and simulated blood loss. It has been suggested that changes in arterial oxygen content are outweighed by opposite changes in cerebral blood flow to maintain cerebral oxygen delivery at or near normal levels [27]. We did not measure arterial partial pressure of oxygen (PaO_2_) in the present trial. However, if we assume an increase in PaO_2_ from 13 to 73 kPa as shown in a similar study [28], that MCAV reflects cerebral blood flow and that SpO_2_ reflects SaO_2_, our data suggest that cerebral oxygen delivery was similar when inhaling oxygen and air (Additional file 4, Figure S2 and S3).

In contrast to the reduction in MCAV with oxygen from baseline to LBNP, there was an increase in ScO_2_ that persisted during LBNP. Consequently, ScO_2_ was elevated at higher LBNP levels when breathing oxygen compared to air. However, it is worth noting that ScO_2_ measured by NIRS captures tissue oxygen saturation only in a region of the anterior brain. In addition, the observed increase with oxygen could be affected by extracranial contamination due to the elevated skin oxygenation. If this indeed reflects a genuine increase in cerebral tissue oxygenation, we either underestimated the increase in CaO_2_ with oxygen, or the changes in MCAV were larger than true cerebral blood flow changes.

Interestingly, we did find that oxygen improved tolerance to simulated blood loss without accompanying changes in the predefined systemic hemodynamic variables. While MCAV decreased with supplemental oxygen, ScO_2_ increased, which could elucidate the improved tolerance to simulated blood loss with oxygen. In our exploratory analyses, we did find a small and statistically significant effect of oxygen on the changes in heart rate during LBNP. One could speculate if the reduction in heart rate could have prolonged the diastole and thereby improved cardiac filling which would fit with the statistically non-significant trend of greater stroke volumes with oxygen.

We observed no effect of oxygen on the changes in SVR and MAP during LBNP, nor did we find any effect from baseline to LBNP. The latter finding appears to diverge from prior research reporting vasoconstriction elevating SVR and MAP during inhalation of 100% oxygen at rest [5]. The suggested rise in SVR with hyperoxia has been proposed to trigger the heart rate reductions, although the underlying mechanism is not well understood [6]. However, it is crucial to note that the present study was neither specifically designed nor adequately powered to examine the isolated effects of oxygen without LBNP, which may account for the disparate findings.

Oxygen therapy in trauma patients is a debated topic and retrospective studies have reported both favorable and unfavorable associations between oxygen and outcome [29]. The TRAUMOX2 trial was initiated due to the lack of randomized trials and hypothesizes that a restrictive compared to a liberal oxygen strategy will improve outcome [30]. We did not observe significant changes in systemic hemodynamics during oxygen therapy that would indicate a disadvantageous effect of oxygen in trauma patients. On the contrary, we found an increased tolerance to simulated blood loss.

While we did not find significant influence of supplemental oxygen on systemic hemodynamics, we did find an effect on cerebral hemodynamics. Future studies should examine whether the observed effect translates to a clinically relevant effect on global or regional cerebral oxygen delivery, e.g., in patients with traumatic brain injury. We observed a reduction in cerebral blood *velocity* with oxygen during LBNP. Whether this reflects lower cerebral blood *flow* under the present conditions remains to be elucidated. Furthermore, future work should investigate whether the increase in arterial oxygen content with hyperoxia counteracts any potential decrease in cerebral blood flow, thereby sustaining adequate oxygen delivery. Based on our finding of improved tolerance to simulated blood loss with oxygen, future studies should also explore if this effect is present in bleeding trauma patients, assess its clinical relevance, and explore possible mechanism.

### Limitations

There are important differences between traumatic and simulated blood loss. In the LBNP model, there is no tissue damage, and the LBNP and oxygen exposure in the present trial had a maximum duration of 24 min, which is less than the typical duration of traumatic blood loss and oxygen exposure, and also not covering a reperfusion phase after volume resuscitation. Consequently, effects that may occur after the acute phase of blood loss are not examined in our study. Also, the present trial only included healthy awake subjects who were breathing spontaneously without concomitant pain or traumatic brain injury which limits the external validity of our study.

Four subjects exhibited hemodynamic decompensation during oxygen visits compared to 10 during air visits, indicating a separation between the two conditions. Since most decompensations during both oxygen and air visits were due to reductions in MAP, there was no evident effect of treatment on the cause of decompensation. Despite decompensation being defined by both subjective (symptoms) and objective (MAP and heart rate) criteria, our results suggest that the majority were driven by objective criteria. Shared frailty models account for random effects and censoring, which is important for our crossover design as LBNP tolerance is reproducible [12]. Consequently, we contend that there exists a credible and objective distinction between oxygen and air.

We employed non-invasive methods in the present trial as they involve low risk in healthy volunteers. Although non-invasive methods in general might be less reliable than invasive methods, we believe they are adequate in the present trial. Stroke volume measured by suprasternal pulsed Doppler ultrasound has been validated previously by Eriksen and Walløe [19]. In brief, blood velocity in the LVOT is maintained with a rectangular (not parabolic) velocity profile for some centimeters into the ascending aorta. Importantly, this velocity is maintained even if the diameter of the sinus of Valsalva and the proximal aorta exceeds that of the LVOT. Consequently, any change in aortic dimensions with LBNP [31] should not affect these measurements. As the ultrasound probe sits well in the suprasternal notch, a fixed angle to the ascending aorta is easy to maintain. The angle of the aorta has been demonstrated to change little with LBNP [31], and a caudal displacement of the heart with LBNP should not be of significance due to the preservation of velocity for the initial centimeters within which the measurements are performed. The diameter of the LVOT was measured only once, as this is believed to be a fixed, fibrous structure [32].

There was a large between-subject variability in cardiac output. Different body sizes, inaccuracies in LVOT measurements and different angles of suprasternal ultrasound insonation may partially explain this. However, these factors would have a minor influence on the results as we analyzed changes from baseline with mixed regression.

Due to large between-subject variability in blood pressure [33] we used a relative reduction in blood pressure, and not an absolute value as a threshold for hemodynamic decompensation. Further, we used MAP rather than systolic pressure as the former determines flow by the Poiseuille equation and thus oxygen delivery.

When calculating mean values for each LBNP level, the values were trimmed for the 5% highest and lowest values to reproducibly and objectively remove outliers caused by, e.g., motion artifacts and extrasystoles. In general, with increased trimming, the mean approaches the median. The level of trimming was largely arbitrarily chosen, but by visual inspection the calculated mean values seemed to fit the observations well. Also, changing the degree of trimming to 0%, 2.5% or 10% did not change the conclusions for the primary outcome.

While our study featured a limited number of subjects, the incorporation of a crossover design, repeated measurements, and a standardized intervention protocol increased the statistical power for each subject. We calculated the sample size to be able to detect, with a reasonable probability, what is considered a clinically relevant difference in the primary outcome cardiac output [22]. We do not believe that a type II error is likely, as the confidence interval does not encompass what we consider a clinically meaningful effect, and we therefore believe the sample size was adequate. The assumptions in the sample size calculation are best judged by the confidence intervals of the estimates [34]. Determining what constitutes a clinically significant effect is, to some extent, subjective and may also be contingent on the context. Importantly, the sample size estimation was performed on the primary outcome, and the study may have been underpowered to detect treatment effects on the secondary and exploratory outcomes.

## Conclusions

In conclusion, the present trial did not demonstrate an effect of oxygen compared to air on the changes in cardiac output during simulated blood loss in healthy volunteers. Neither did we find an effect on the changes in stroke volume or MCAV during simulated blood loss. We did however find an increased tolerance to simulated blood loss with oxygen, that was accompanied by elevated ScO_2_ in our exploratory analyses. The exploratory analyses also revealed a less pronounced elevation in heart rate with oxygen during simulated blood loss. Our findings suggest that supplemental oxygen does not adversely affect the systemic and cerebral hemodynamic response to simulated blood loss.

### Supplementary Information

**Additional file1. **Original protocol.

**Additional file 2.** CONSORT checklist.

**Additional file 3.** Data for conducting analyses for the primary and secondary outcomes.

**Additional file 4.** Regression outputs for the reported results.

**Additional file 5.** R script with code used for the primary and secondary outcomes.

## Data Availability

Data for the prespecified outcomes are provided in Additional file 3. R code for analyses is provided in Additional file 5.

## Abbreviations

LBNP: Lower body negative pressure
LVOT: Left ventricular outflow tract
MCAV: Middle cerebral artery blood velocity
MAP: Mean arterial pressure
ScO_2_: Cerebral tissue oxygen saturation
SVR: Systemic vascular resistance
SpO_2_: Peripheral oxygen saturation
SD: Standard deviation
CI: Confidence interval
