# Supplementary material for: Hemodynamic effects of supplemental oxygen versus air in simulated blood loss in healthy volunteers: a randomized, controlled, double-blind, crossover trial
Source: Intensive Care Med Exp. 2023 Nov 10;11:76. doi: 10.1186/s40635-023-00561-z (PMC10638149; doi:10.1186/s40635-023-00561-z)
Supplement: Supplementary file 4 — Additional file 4. Regression outputs for the reported results. [file 40635_2023_561_MOESM4_ESM.docx]

Regression outputs for “Hemodynamic effects of supplemental oxygen versus air in simulated blood loss in healthy volunteers: A randomized, controlled, double-blind, crossover trial”

2023-09-11

Regression modelling

In this additional file we provide the regression models for the reported results in the published paper. The effect of oxygen compared to air on the absolute changes in the outcome variables during lower body negative pressure (LBNP) was analyzed with mixed linear regression with subjects as a random effect (intercept) to account for repeated measurements within subjects using the “lme” function of the “nlme” package. The outcome variable was entered as the response variable while LBNP level and treatment, including their interaction effects, were entered as explanatory variables. The interaction term of LBNP level and treatment was considered the treatment specific effect on the response variable. The main effect of treatment was considered the effect from baseline to LBNP. We compared this parsimonious model to models including polynomial terms of LBNP level up to power two, including an interaction with treatment, to account for a potential non-linear change in the response variable with LBNP using Aikaike information criterion. LBNP level was treated as a continuous variable. Treatment was entered as a factor. Statistically non-significant polynomial terms and interactions with treatment were removed from the model.

Tolerance to simulated blood loss was analysed using survival analysis in a shared frailty model with time to hemodynamic decompensation measured as the time from the start of LBNP 0 until completing LBNP 80 or stopping. Censoring occurred when finishing LBNP 80. Subjects were entered as a random effect.

Precisions in MCAV and stroke volume measurements were calculated on baseline data at rest without treatment. The entire baseline period of 3 minutes was divided into 1 minute averages per subject per visit and analyzed in a mixed linear regression model with subjects, and visits nested within subjects, as a random intercept. Precision was calculated as 1.96 × the residual SD.

SpO_2_


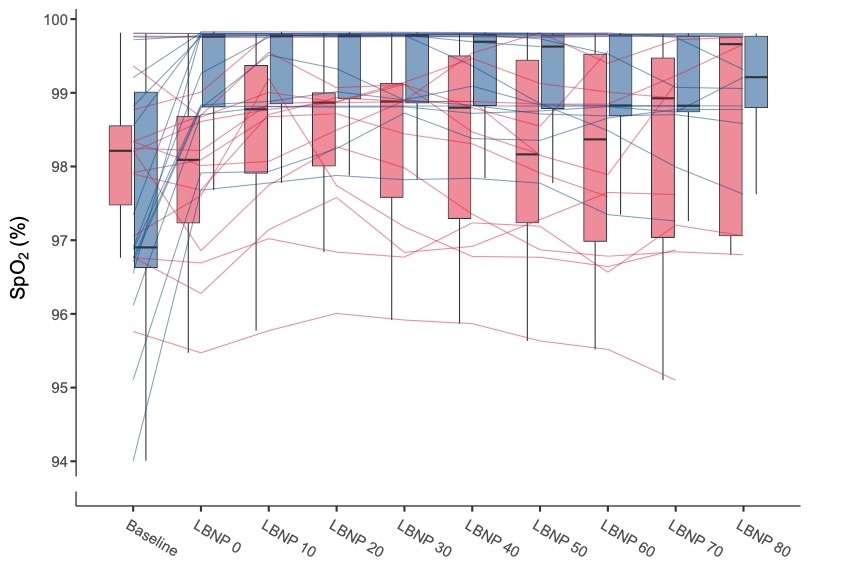


Figure S1. Peripheral oxygen saturation (SpO_2_) during lower body negative pressure (LBNP) from 0 to 80 mmHg with oxygen (blue) and air (red). Thin lines represent subjects. Boxplot with median, 25^th^ and 75^th^ percentiles and whiskers as 1.5 x interquartile range.

Cardiac output

Model 1 – reported model

|  | **Cardiac output** | | | |
| --- | --- | --- | --- | --- |
| *Predictors* | *Estimates* | *SE* | *95% CI* | *p-val* |
| (Intercept) | -0.1120 | 0.1248 | -0.3580 to 0.1339 | 0.370 |
| LBNP_level | -0.2240 | 0.0174 | -0.2583 to -0.1897 | **<0.001** |
| Treatment [Oxygen] | -0.1021 | 0.1007 | -0.3006 to 0.0963 | 0.312 |
| LBNP_level * Treatment [Oxygen] | 0.0310 | 0.0235 | -0.0152 to 0.0773 | 0.188 |
| **Random Effects** | | | | |
| σ^2^ | 0.19 | | | |
| τ_00_ _ID_ | 0.16 | | | |
| ICC | 0.45 | | | |
| N _ID_ | 15 | | | |
| Observations | 240 | | | |
| Marginal R^2^ / Conditional R^2^ | 0.422 / 0.683 | | | |
| AIC | 348.543 | | | |

Model 2

|  | **Cardiac output** | | | |
| --- | --- | --- | --- | --- |
| *Predictors* | *Estimates* | *SE* | *95% CI* | *p-val* |
| (Intercept) | -0.1100 | 0.1309 | -0.3680 to 0.1479 | 0.401 |
| LBNP_level | -0.2259 | 0.0413 | -0.3074 to -0.1444 | **<0.001** |
| Treatment [Oxygen] | -0.1019 | 0.1011 | -0.3011 to 0.0973 | 0.315 |
| LBNP_level^2 | 0.0003 | 0.0052 | -0.0100 to 0.0106 | 0.960 |
| LBNP_level * Treatment [Oxygen] | 0.0309 | 0.0237 | -0.0157 to 0.0776 | 0.193 |
| **Random Effects** | | | | |
| σ^2^ | 0.19 | | | |
| τ_00_ _ID_ | 0.16 | | | |
| ICC | 0.45 | | | |
| N _ID_ | 15 | | | |
| Observations | 240 | | | |
| Marginal R^2^ / Conditional R^2^ | 0.421 / 0.682 | | | |
| AIC | 359.212 | | | |

Model 3

|  | **Cardiac output** | | | |
| --- | --- | --- | --- | --- |
| *Predictors* | *Estimates* | *SE* | *95% CI* | *p-val* |
| (Intercept) | -0.1151 | 0.1382 | -0.3876 to 0.1573 | 0.406 |
| LBNP_level | -0.2211 | 0.0592 | -0.3378 to -0.1044 | **<0.001** |
| Treatment [Oxygen] | -0.0923 | 0.1315 | -0.3514 to 0.1668 | 0.483 |
| LBNP_level^2 | -0.0004 | 0.0079 | -0.0159 to 0.0151 | 0.959 |
| LBNP_level * Treatment [Oxygen] | 0.0220 | 0.0812 | -0.1380 to 0.1821 | 0.786 |
| Treatment [Oxygen] * LBNP_level^2 | 0.0012 | 0.0105 | -0.0195 to 0.0219 | 0.909 |
| **Random Effects** | | | | |
| σ^2^ | 0.19 | | | |
| τ_00_ _ID_ | 0.16 | | | |
| ICC | 0.45 | | | |
| N _ID_ | 15 | | | |
| Observations | 240 | | | |
| Marginal R^2^ / Conditional R^2^ | 0.420 / 0.681 | | | |
| AIC | 368.479 | | | |

Stroke volume

Model 4 - reported model

|  | **Stroke volume** | | | |
| --- | --- | --- | --- | --- |
| *Predictors* | *Estimates* | *SE* | *95% CI* | *p-val* |
| (Intercept) | 1.1499 | 1.7767 | -2.3514 to 4.6512 | 0.518 |
| LBNP_level | -6.8076 | 0.2925 | -7.3840 to -6.2311 | **<0.001** |
| Treatment [Oxygen] | 2.4196 | 1.6935 | -0.9177 to 5.7569 | 0.154 |
| LBNP_level * Treatment [Oxygen] | 0.3851 | 0.3949 | -0.3932 to 1.1634 | 0.331 |
| **Random Effects** | | | | |
| σ^2^ | 53.53 | | | |
| τ_00_ _ID_ | 25.30 | | | |
| N _ID_ | 15 | | | |
| Observations | 240 | | | |
| Marginal R^2^ / Conditional R^2^ | 0.826 / NA | | | |
| AIC | 1673.643 | | | |

Model 5

|  | **Stroke volume** | | | |
| --- | --- | --- | --- | --- |
| *Predictors* | *Estimates* | *SE* | *95% CI* | *p-val* |
| (Intercept) | 0.3083 | 1.8894 | -3.4152 to 4.0318 | 0.871 |
| LBNP_level | -6.0000 | 0.6926 | -7.3649 to -4.6351 | **<0.001** |
| Treatment [Oxygen] | 2.3061 | 1.6936 | -1.0316 to 5.6438 | 0.175 |
| LBNP_level^2 | -0.1126 | 0.0876 | -0.2853 to 0.0600 | 0.200 |
| LBNP_level * Treatment [Oxygen] | 0.4361 | 0.3964 | -0.3452 to 1.2174 | 0.272 |
| **Random Effects** | | | | |
| σ^2^ | 53.39 | | | |
| τ_00_ _ID_ | 25.12 | | | |
| N _ID_ | 15 | | | |
| Observations | 240 | | | |
| Marginal R^2^ / Conditional R^2^ | 0.827 / NA | | | |
| AIC | 1677.023 | | | |

Model 6

|  | **Stroke volume** | | | |
| --- | --- | --- | --- | --- |
| *Predictors* | *Estimates* | *SE* | *95% CI* | *p-val* |
| (Intercept) | 0.0698 | 2.0309 | -3.9328 to 4.0724 | 0.973 |
| LBNP_level | -5.7720 | 0.9918 | -7.7266 to -3.8174 | **<0.001** |
| Treatment [Oxygen] | 2.7576 | 2.2025 | -1.5830 to 7.0983 | 0.212 |
| LBNP_level^2 | -0.1443 | 0.1318 | -0.4041 to 0.1155 | 0.275 |
| LBNP_level * Treatment [Oxygen] | 0.0174 | 1.3607 | -2.6642 to 2.6991 | 0.990 |
| Treatment [Oxygen] * LBNP_level^2 | 0.0565 | 0.1756 | -0.2896 to 0.4026 | 0.748 |
| **Random Effects** | | | | |
| σ^2^ | 53.61 | | | |
| τ_00_ _ID_ | 25.09 | | | |
| N _ID_ | 15 | | | |
| Observations | 240 | | | |
| Marginal R^2^ / Conditional R^2^ | 0.827 / NA | | | |
| AIC | 1680.562 | | | |

Middle cerebral artery blood velocity (MCAV)

Model 7 - reported model

|  | **MCAV** | | | |
| --- | --- | --- | --- | --- |
| *Predictors* | *Estimates* | *SE* | *95% CI* | *p-val* |
| (Intercept) | 0.7494 | 1.3397 | -1.8909 to 3.3897 | 0.576 |
| LBNP_level | -1.0731 | 0.1381 | -1.3453 to -0.8010 | **<0.001** |
| Treatment [Oxygen] | -5.2362 | 0.8020 | -6.8167 to -3.6557 | **<0.001** |
| LBNP_level * Treatment [Oxygen] | 0.2480 | 0.1828 | -0.1123 to 0.6084 | 0.176 |
| **Random Effects** | | | | |
| σ^2^ | 11.64 | | | |
| τ_00_ _ID_ | 21.76 | | | |
| N _ID_ | 15 | | | |
| Observations | 238 | | | |
| Marginal R^2^ / Conditional R^2^ | 0.477 / NA | | | |
| AIC | 1320.646 | | | |

Model 8

|  | **MCAV** | | | |
| --- | --- | --- | --- | --- |
| *Predictors* | *Estimates* | *SE* | *95% CI* | *p-val* |
| (Intercept) | 0.4812 | 1.3739 | -2.2265 to 3.1890 | 0.726 |
| LBNP_level | -0.8196 | 0.3234 | -1.4570 to -0.1821 | **0.012** |
| Treatment [Oxygen] | -5.2741 | 0.8036 | -6.8580 to -3.6902 | **<0.001** |
| LBNP_level^2 | -0.0348 | 0.0401 | -0.1139 to 0.0443 | 0.387 |
| LBNP_level * Treatment [Oxygen] | 0.2646 | 0.1839 | -0.0979 to 0.6272 | 0.152 |
| **Random Effects** | | | | |
| σ^2^ | 11.65 | | | |
| τ_00_ _ID_ | 21.70 | | | |
| N _ID_ | 15 | | | |
| Observations | 238 | | | |
| Marginal R^2^ / Conditional R^2^ | 0.478 / NA | | | |
| AIC | 1326.488 | | | |

Model 9

|  | **MCAV** | | | |
| --- | --- | --- | --- | --- |
| *Predictors* | *Estimates* | *SE* | *95% CI* | *p-val* |
| (Intercept) | 0.2646 | 1.4217 | -2.5374 to 3.0666 | 0.853 |
| LBNP_level | -0.6165 | 0.4698 | -1.5424 to 0.3094 | 0.191 |
| Treatment [Oxygen] | -4.8756 | 1.0459 | -6.9369 to -2.8143 | **<0.001** |
| LBNP_level^2 | -0.0626 | 0.0615 | -0.1838 to 0.0586 | 0.310 |
| LBNP_level * Treatment [Oxygen] | -0.0985 | 0.6357 | -1.3514 to 1.1544 | 0.877 |
| Treatment [Oxygen] * LBNP_level^2 | 0.0483 | 0.0809 | -0.1112 to 0.2078 | 0.551 |
| **Random Effects** | | | | |
| σ^2^ | 11.69 | | | |
| τ_00_ _ID_ | 21.71 | | | |
| N _ID_ | 15 | | | |
| Observations | 238 | | | |
| Marginal R^2^ / Conditional R^2^ | 0.477 / NA | | | |
| AIC | 1331.323 | | | |

Tolerance to simulated blood loss

Model 10

## Cox mixed-effects model fit by maximum likelihood

## Data: df_surv

## events, n = 14, 30

## Iterations= 10 54

## NULL Integrated Fitted

## Log-likelihood -43.98638 -40.33183 -29.16928

##

## Chisq df p AIC BIC

## Integrated loglik 7.31 2.00 0.02587300 3.31 2.03

## Penalized loglik 29.63 9.81 0.00086778 10.02 3.76

##

## Model: Surv(Survival_time, Status) ~ Treatment + (1 | ID)

## Fixed coefficients

## coef exp(coef) se(coef) z p

## TreatmentOxygen -1.447246 0.2352171 0.6554491 -2.21 0.027

##

## Random effects

## Group Variable Std Dev Variance

## ID Intercept 1.470769 2.163162

Explorative regression analyses

Heart rate

Model 11

|  | **Heart rate** | | | |
| --- | --- | --- | --- | --- |
| *Predictors* | *Estimates* | *SE* | *95% CI* | *p-val* |
| (Intercept) | -7.5168 | 1.8601 | -11.1819 to -3.8518 | **<0.001** |
| LBNP_level | 6.1384 | 0.3434 | 5.4618 to 6.8151 | **<0.001** |
| Treatment [Oxygen] | -3.1809 | 2.0382 | -7.1969 to 0.8351 | 0.120 |
| LBNP_level * Treatment [Oxygen] | -0.5230 | 0.4638 | -1.4370 to 0.3909 | 0.261 |
| **Random Effects** | | | | |
| σ^2^ | 78.66 | | | |
| τ_00_ _ID_ | 19.97 | | | |
| N _ID_ | 15 | | | |
| Observations | 247 | | | |
| Marginal R^2^ / Conditional R^2^ | 0.728 / NA | | | |
| AIC | 1808.095 | | | |

Model 12 – reported model

|  | **Heart rate** | | | |
| --- | --- | --- | --- | --- |
| *Predictors* | *Estimates* | *SE* | *95% CI* | *p-val* |
| (Intercept) | -1.3072 | 1.9092 | -5.0691 to 2.4547 | 0.494 |
| LBNP_level | 0.2837 | 0.7059 | -1.1072 to 1.6746 | 0.688 |
| Treatment [Oxygen] | -2.3328 | 1.7456 | -5.7724 to 1.1068 | 0.183 |
| LBNP_level^2 | 0.8029 | 0.0879 | 0.6298 to 0.9760 | **<0.001** |
| LBNP_level * Treatment [Oxygen] | -0.8991 | 0.3989 | -1.6850 to -0.1132 | **0.025** |
| **Random Effects** | | | | |
| σ^2^ | 57.53 | | | |
| τ_00_ _ID_ | 24.33 | | | |
| N _ID_ | 15 | | | |
| Observations | 247 | | | |
| Marginal R^2^ / Conditional R^2^ | 0.802 / NA | | | |
| AIC | 1742.132 | | | |

Model 13

|  | **Heart rate** | | | |
| --- | --- | --- | --- | --- |
| *Predictors* | *Estimates* | *SE* | *95% CI* | *p-val* |
| (Intercept) | -1.0396 | 2.0614 | -5.1016 to 3.0224 | 0.615 |
| LBNP_level | 0.0325 | 1.0113 | -1.9602 to 2.0253 | 0.974 |
| Treatment [Oxygen] | -2.8403 | 2.2785 | -7.3300 to 1.6494 | 0.214 |
| LBNP_level^2 | 0.8372 | 0.1322 | 0.5767 to 1.0977 | **<0.001** |
| LBNP_level * Treatment [Oxygen] | -0.4369 | 1.3890 | -3.1739 to 2.3001 | 0.753 |
| Treatment [Oxygen] * LBNP_level^2 | -0.0613 | 0.1765 | -0.4092 to 0.2865 | 0.729 |
| **Random Effects** | | | | |
| σ^2^ | 57.75 | | | |
| τ_00_ _ID_ | 24.39 | | | |
| N _ID_ | 15 | | | |
| Observations | 247 | | | |
| Marginal R^2^ / Conditional R^2^ | 0.801 / NA | | | |
| AIC | 1745.644 | | | |

Mean arterial blood pressure (MAP)

Model 14 - reported model

|  | **MAP** | | | |
| --- | --- | --- | --- | --- |
| *Predictors* | *Estimates* | *SE* | *95% CI* | *p-val* |
| (Intercept) | 2.0259 | 0.9387 | 0.1763 to 3.8756 | **0.032** |
| LBNP_level | -0.5925 | 0.1418 | -0.8719 to -0.3130 | **<0.001** |
| Treatment [Oxygen] | 1.3082 | 0.8412 | -0.3493 to 2.9656 | 0.121 |
| LBNP_level * Treatment [Oxygen] | 0.0720 | 0.1915 | -0.3053 to 0.4493 | 0.707 |
| **Random Effects** | | | | |
| σ^2^ | 13.40 | | | |
| τ_00_ _ID_ | 7.78 | | | |
| N _ID_ | 15 | | | |
| Observations | 247 | | | |
| Marginal R^2^ / Conditional R^2^ | 0.150 / NA | | | |
| AIC | 1387.905 | | | |

Model 15

|  | **MAP** | | | |
| --- | --- | --- | --- | --- |
| *Predictors* | *Estimates* | *SE* | *95% CI* | *p-val* |
| (Intercept) | 1.4468 | 0.9901 | -0.5042 to 3.3977 | 0.145 |
| LBNP_level | -0.0474 | 0.3392 | -0.7157 to 0.6210 | 0.889 |
| Treatment [Oxygen] | 1.2304 | 0.8387 | -0.4222 to 2.8830 | 0.144 |
| LBNP_level^2 | -0.0746 | 0.0422 | -0.1578 to 0.0086 | 0.078 |
| LBNP_level * Treatment [Oxygen] | 0.1066 | 0.1917 | -0.2711 to 0.4842 | 0.579 |
| **Random Effects** | | | | |
| σ^2^ | 13.28 | | | |
| τ_00_ _ID_ | 7.70 | | | |
| N _ID_ | 15 | | | |
| Observations | 247 | | | |
| Marginal R^2^ / Conditional R^2^ | 0.161 / NA | | | |
| AIC | 1391.281 | | | |

Model 16

|  | **MAP** | | | |
| --- | --- | --- | --- | --- |
| *Predictors* | *Estimates* | *SE* | *95% CI* | *p-val* |
| (Intercept) | 1.5903 | 1.0583 | -0.4951 to 3.6756 | 0.134 |
| LBNP_level | -0.1820 | 0.4859 | -1.1395 to 0.7755 | 0.708 |
| Treatment [Oxygen] | 0.9583 | 1.0947 | -1.1987 to 3.1153 | 0.382 |
| LBNP_level^2 | -0.0562 | 0.0635 | -0.1814 to 0.0689 | 0.377 |
| LBNP_level * Treatment [Oxygen] | 0.3544 | 0.6674 | -0.9606 to 1.6694 | 0.596 |
| Treatment [Oxygen] * LBNP_level^2 | -0.0329 | 0.0848 | -0.2000 to 0.1342 | 0.699 |
| **Random Effects** | | | | |
| σ^2^ | 13.33 | | | |
| τ_00_ _ID_ | 7.72 | | | |
| N _ID_ | 15 | | | |
| Observations | 247 | | | |
| Marginal R^2^ / Conditional R^2^ | 0.161 / NA | | | |
| AIC | 1396.229 | | | |

Systemic vascular resistance (SVR)

Model 17 - reported model

|  | **SVR** | | | |
| --- | --- | --- | --- | --- |
| *Predictors* | *Estimates* | *SE* | *95% CI* | *p-val* |
| (Intercept) | 0.3951 | 1.4254 | -2.4139 to 3.2042 | 0.782 |
| LBNP_level | 1.7141 | 0.2045 | 1.3110 to 2.1171 | **<0.001** |
| Treatment [Oxygen] | 0.8049 | 1.1836 | -1.5275 to 3.1373 | 0.497 |
| LBNP_level * Treatment [Oxygen] | 0.0708 | 0.2761 | -0.4732 to 0.6149 | 0.798 |
| **Random Effects** | | | | |
| σ^2^ | 26.14 | | | |
| τ_00_ _ID_ | 19.70 | | | |
| N _ID_ | 15 | | | |
| Observations | 240 | | | |
| Marginal R^2^ / Conditional R^2^ | 0.413 / NA | | | |
| AIC | 1510.433 | | | |

Model 18

|  | **SVR** | | | |
| --- | --- | --- | --- | --- |
| *Predictors* | *Estimates* | *SE* | *95% CI* | *p-val* |
| (Intercept) | 0.4145 | 1.4986 | -2.5388 to 3.3677 | 0.782 |
| LBNP_level | 1.6956 | 0.4858 | 0.7381 to 2.6530 | **0.001** |
| Treatment [Oxygen] | 0.8074 | 1.1878 | -1.5335 to 3.1483 | 0.497 |
| LBNP_level^2 | 0.0026 | 0.0615 | -0.1185 to 0.1237 | 0.967 |
| LBNP_level * Treatment [Oxygen] | 0.0697 | 0.2781 | -0.4783 to 0.6177 | 0.802 |
| **Random Effects** | | | | |
| σ^2^ | 26.26 | | | |
| τ_00_ _ID_ | 19.70 | | | |
| N _ID_ | 15 | | | |
| Observations | 240 | | | |
| Marginal R^2^ / Conditional R^2^ | 0.412 / NA | | | |
| AIC | 1516.174 | | | |

Model 19

|  | **SVR** | | | |
| --- | --- | --- | --- | --- |
| *Predictors* | *Estimates* | *SE* | *95% CI* | *p-val* |
| (Intercept) | 0.1676 | 1.5858 | -2.9576 to 3.2929 | 0.916 |
| LBNP_level | 1.9315 | 0.6955 | 0.5608 to 3.3023 | **0.006** |
| Treatment [Oxygen] | 1.2746 | 1.5444 | -1.7691 to 4.3182 | 0.410 |
| LBNP_level^2 | -0.0302 | 0.0925 | -0.2124 to 0.1520 | 0.744 |
| LBNP_level * Treatment [Oxygen] | -0.3635 | 0.9542 | -2.2440 to 1.5170 | 0.704 |
| Treatment [Oxygen] * LBNP_level^2 | 0.0585 | 0.1232 | -0.1843 to 0.3012 | 0.635 |
| **Random Effects** | | | | |
| σ^2^ | 26.36 | | | |
| τ_00_ _ID_ | 19.63 | | | |
| N _ID_ | 15 | | | |
| Observations | 240 | | | |
| Marginal R^2^ / Conditional R^2^ | 0.411 / NA | | | |
| AIC | 1520.301 | | | |

Cerebral tissue oxygen saturation (ScO_2_)

Model 20 - reported model

|  | **ScO2** | | | |
| --- | --- | --- | --- | --- |
| *Predictors* | *Estimates* | *SE* | *95% CI* | *p-val* |
| (Intercept) | 0.6021 | 0.6170 | -0.6140 to 1.8182 | 0.330 |
| LBNP_level | -0.8091 | 0.1140 | -1.0338 to -0.5845 | **<0.001** |
| Treatment [Oxygen] | 4.9547 | 0.6740 | 3.6262 to 6.2832 | **<0.001** |
| LBNP_level * Treatment [Oxygen] | 0.1724 | 0.1520 | -0.1271 to 0.4719 | 0.258 |
| **Random Effects** | | | | |
| σ^2^ | 7.85 | | | |
| τ_00_ _ID_ | 2.22 | | | |
| N _ID_ | 15 | | | |
| Observations | 232 | | | |
| Marginal R^2^ / Conditional R^2^ | 0.566 / NA | | | |
| AIC | 1174.474 | | | |

Model 21

|  | **ScO2** | | | |
| --- | --- | --- | --- | --- |
| *Predictors* | *Estimates* | *SE* | *95% CI* | *p-val* |
| (Intercept) | 0.3545 | 0.6677 | -0.9616 to 1.6706 | 0.596 |
| LBNP_level | -0.5732 | 0.2688 | -1.1030 to -0.0433 | **0.034** |
| Treatment [Oxygen] | 4.9089 | 0.6757 | 3.5769 to 6.2409 | **<0.001** |
| LBNP_level^2 | -0.0326 | 0.0336 | -0.0989 to 0.0337 | 0.334 |
| LBNP_level * Treatment [Oxygen] | 0.1917 | 0.1533 | -0.1104 to 0.4938 | 0.212 |
| **Random Effects** | | | | |
| σ^2^ | 7.85 | | | |
| τ_00_ _ID_ | 2.22 | | | |
| N _ID_ | 15 | | | |
| Observations | 232 | | | |
| Marginal R^2^ / Conditional R^2^ | 0.567 / NA | | | |
| AIC | 1180.482 | | | |

Model 22

|  | **ScO2** | | | |
| --- | --- | --- | --- | --- |
| *Predictors* | *Estimates* | *SE* | *95% CI* | *p-val* |
| (Intercept) | 0.2636 | 0.7324 | -1.1801 to 1.7073 | 0.719 |
| LBNP_level | -0.4870 | 0.3911 | -1.2579 to 0.2838 | 0.214 |
| Treatment [Oxygen] | 5.0779 | 0.8760 | 3.3510 to 6.8048 | **<0.001** |
| LBNP_level^2 | -0.0445 | 0.0516 | -0.1463 to 0.0573 | 0.390 |
| LBNP_level * Treatment [Oxygen] | 0.0367 | 0.5329 | -1.0138 to 1.0871 | 0.945 |
| Treatment [Oxygen] * LBNP_level^2 | 0.0207 | 0.0680 | -0.1134 to 0.1547 | 0.762 |
| **Random Effects** | | | | |
| σ^2^ | 7.88 | | | |
| τ_00_ _ID_ | 2.22 | | | |
| N _ID_ | 15 | | | |
| Observations | 232 | | | |
| Marginal R^2^ / Conditional R^2^ | 0.566 / NA | | | |
| AIC | 1185.930 | | | |

Precision in MCAV and stroke volume calculated using mixed regression

Precision in MCAV

|  | **Precision in MCAV** | | | |
| --- | --- | --- | --- | --- |
| *Predictors* | *Estimates* | *SE* | *95% CI* | *p-val* |
| (Intercept) | 59.5477 | 3.5130 | 52.5157 to 66.5797 | **<0.001** |
| **Random Effects** | | | | |
| σ^2^ | 4.44 | | | |
| τ_00_ _Visit_ | 12.81 | | | |
| τ_00_ _ID_ | 6.19 | | | |
| N _Visit_ | 2 | | | |
| N _ID_ | 15 | | | |
| Observations | 87 | | | |
| Marginal R^2^ / Conditional R^2^ | 0.000 / NA | | | |
| AIC | 507.467 | | | |

The precision in MCAV was sqrt(4.44) * 1.96 = 4.1 cm/s.

Precision in stroke volume

|  | **Precision in stroke volume** | | | |
| --- | --- | --- | --- | --- |
| *Predictors* | *Estimates* | *SE* | *95% CI* | *p-val* |
| (Intercept) | 74.2314 | 4.1626 | 65.9050 to 82.5578 | **<0.001** |
| **Random Effects** | | | | |
| σ^2^ | 7.90 | | | |
| τ_00_ _Visit_ | 14.88 | | | |
| τ_00_ _ID_ | 8.63 | | | |
| N _Visit_ | 2 | | | |
| N _ID_ | 15 | | | |
| Observations | 90 | | | |
| Marginal R^2^ / Conditional R^2^ | 0.000 / NA | | | |
| AIC | 573.708 | | | |

The precision in stroke volume was sqrt(7.90) * 1.96 = 5.5 mL.

The effect of supplemental oxygen on arterial oxygen content and cerebral oxygen delivery

Arterial oxygen content (CaO_2_)

Assumptions:

- PaO_2_ when breathing air: 97 mmHg (13 kpa)
- PaO_2_ when breathing oxygen: 548 mmHg (73 kPa)
- Hemoglobin for women: 14 g/dL
- Hemoglobin for men: 15 g/dL
- SpO_2_ reflects SaO_2_

CaO_2_ = 1.36 x Hb x SpO_2_/100 + 0.003 x PaO_2_

|  | **CaO_2_** | | | |
| --- | --- | --- | --- | --- |
| *Predictors* | *Estimates* | *SE* | *95% CI* | *p-val* |
| (Intercept) | 0.0491 | 0.0447 | -0.0391 to 0.1372 | 0.274 |
| LBNP_level | -0.0008 | 0.0059 | -0.0124 to 0.0108 | 0.890 |
| Treatment [Oxygen] | 1.6735 | 0.0349 | 1.6047 to 1.7422 | **<0.001** |
| LBNP_level * Treatment [Oxygen] | -0.0015 | 0.0079 | -0.0172 to 0.0141 | 0.850 |
| **Random Effects** | | | | |
| σ^2^ | 0.02 | | | |
| τ_00_ _ID_ | 0.02 | | | |
| ICC | 0.47 | | | |
| N _ID_ | 15 | | | |
| Observations | 247 | | | |
| Marginal R^2^ / Conditional R^2^ | 0.941 / 0.969 | | | |
| AIC | -153.182 | | | |


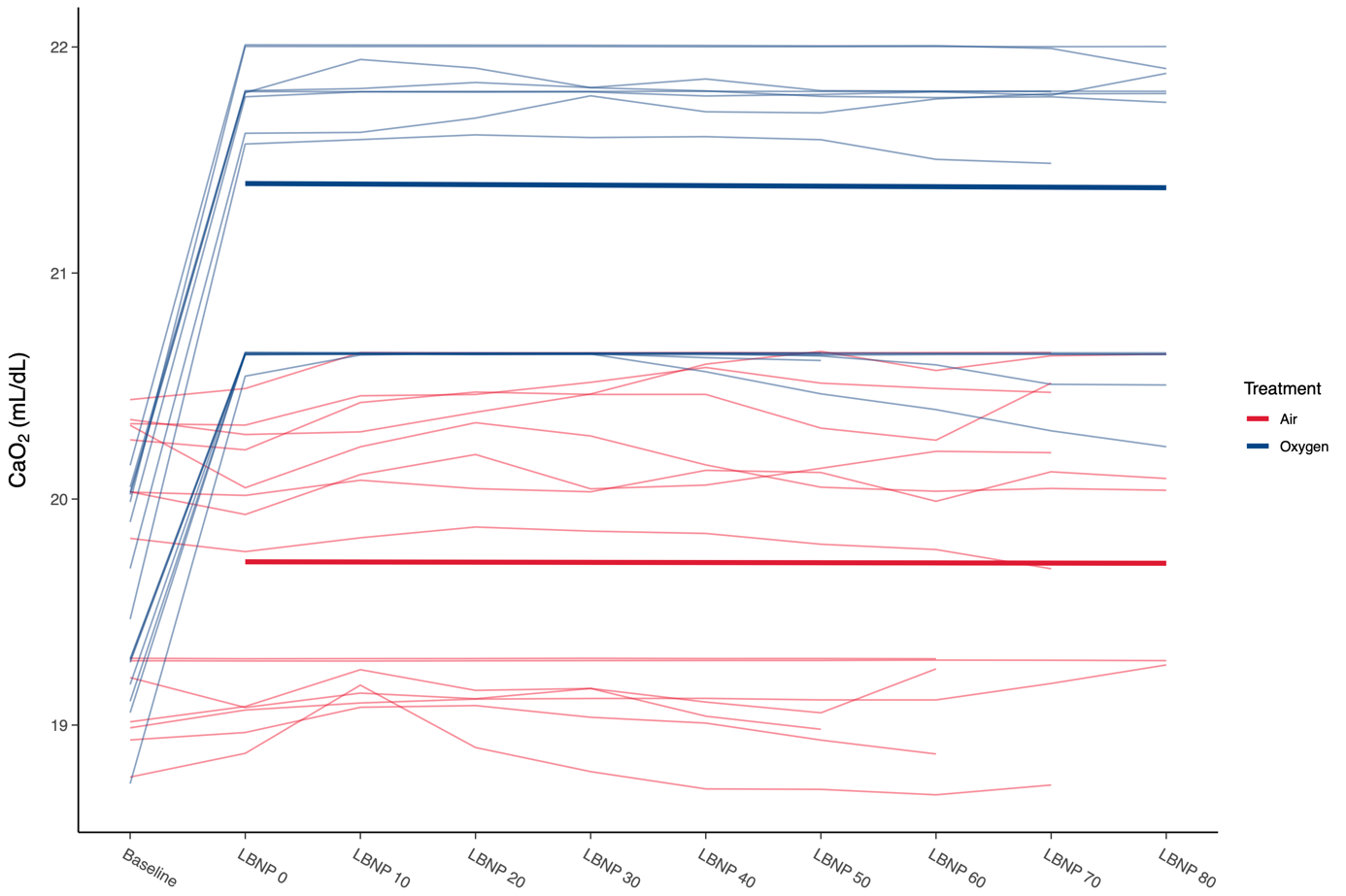


Figure S2. Estimated arterial oxygen content (CaO_2_) during lower body negative pressure (LBNP) from 0 to 80 mmHg with oxygen (blue) and air (red). Thin lines represent subjects. Thick lines are regression estimates of the effect of oxygen and air on the changes in CaO_2_ during LBNP.

Cerebral oxygen delivery (DO_2_)

Assumptions:

- MCAV reflects cerebral blood flow

Cerebral DO_2_ = MCAV x CaO_2_

|  | **Cerebral DO_2_** | | | |
| --- | --- | --- | --- | --- |
| *Predictors* | *Estimates* | *SE* | *95% CI* | *p-val* |
| (Intercept) | 19.0664 | 25.4312 | -31.0536 to 69.1864 | 0.454 |
| LBNP_level | -21.2234 | 2.8834 | -26.9061 to -15.5407 | **<0.001** |
| Treatment [Oxygen] | -11.7851 | 16.7454 | -44.7871 to 21.2169 | 0.482 |
| LBNP_level * Treatment [Oxygen] | 3.3506 | 3.8178 | -4.1736 to 10.8748 | 0.381 |
| **Random Effects** | | | | |
| σ^2^ | 5074.31 | | | |
| τ_00_ _ID_ | 7448.67 | | | |
| N _ID_ | 15 | | | |
| Observations | 238 | | | |
| Marginal R^2^ / Conditional R^2^ | 0.309 / NA | | | |
| AIC | 2739.592 | | | |


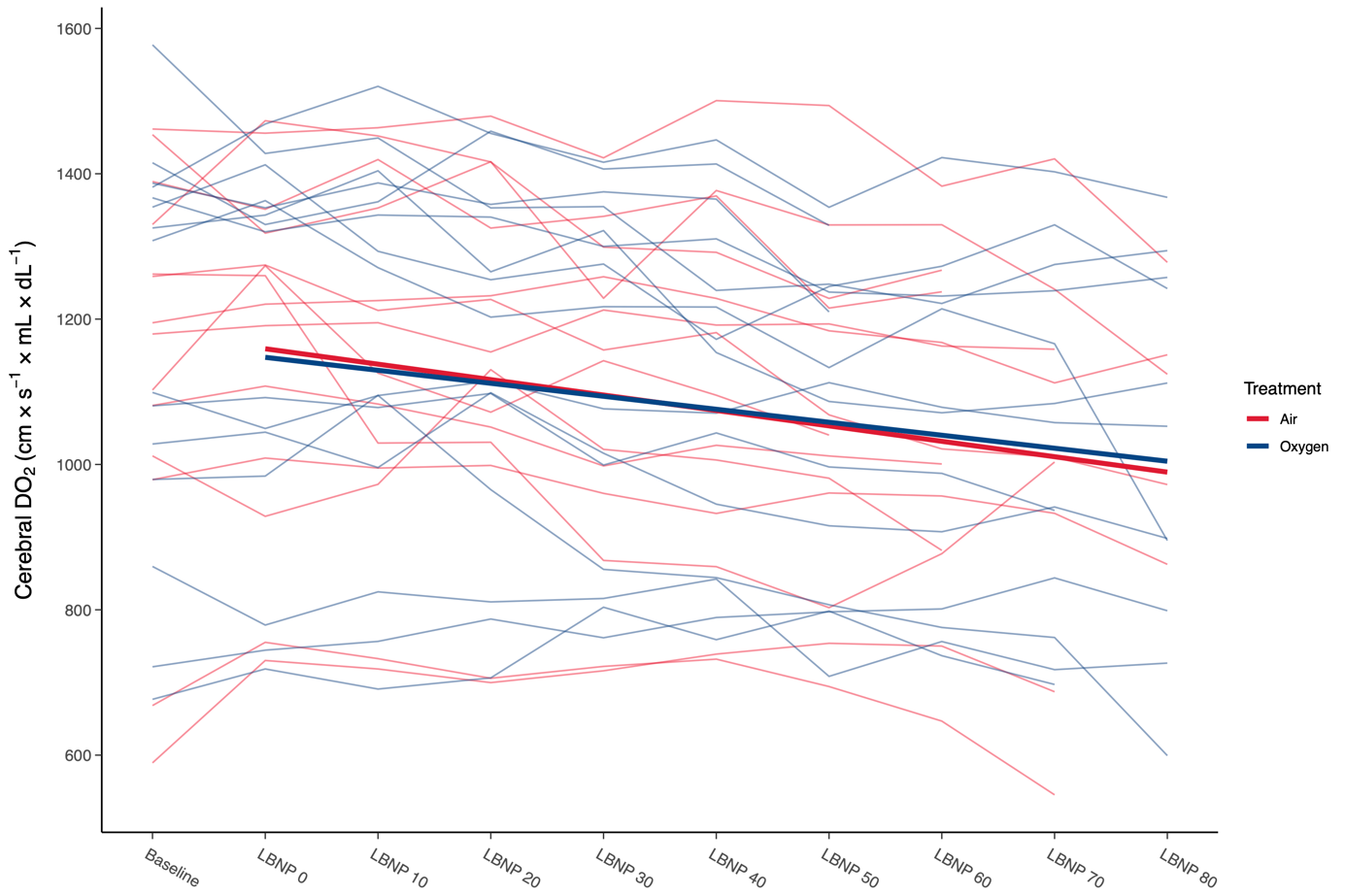


Figure S3. Estimated cerebral oxygen delivery (DO_2_) during lower body negative pressure (LBNP) from 0 to 80 mmHg with oxygen (blue) and air (red). Thin lines represent subjects. Thick lines are regression estimates of the effect of oxygen and air on the changes in cerebral DO_2_ during LBNP.
