# Supplementary material for: Hemodynamic effects of supplemental oxygen versus air in simulated blood loss in healthy volunteers: a randomized, controlled, double-blind, crossover trial
Source: Intensive Care Med Exp. 2023 Nov 10;11:76. doi: 10.1186/s40635-023-00561-z (PMC10638149; doi:10.1186/s40635-023-00561-z)
Supplement: Supplementary file 5 — Additional file 5. R script with code used for the primary and secondary outcomes. [file 40635_2023_561_MOESM5_ESM.docx]

########################################################################################## Analyses of prespecified outcomes #####################

######################################################################

# This is a R script for the trial "Hemodynamic effects of supplemental oxygen versus air in simulated

# blood loss in healthy volunteers: A randomized, controlled, double-blind, crossover trial" containing

# code to perform analyses for the primary and secondary outcomes.

# Open the shared dataset.

df <- read.csv() %>% # Enter file name and file location.

mutate(Treatment = factor(Treatment, levels = c("Air", "Oxygen")))

View(df)

# Primary outcome - cardiac output ----------------------------------------

lme.co <- lme(Cardiac_output_change ~ LBNP_level + LBNP_level*Treatment,

random = ~ 1|Subject,

na.action = na.omit,

data = df)

summary(lme.co)

tab_model(lme.co)

# Secondary outcomes ----------------------------------------

## Stroke volume

lme.sv <- lme(Stroke_volume_change ~ LBNP_level + LBNP_level*Treatment,

random = ~ 1|Subject,

na.action = na.omit,

data = df)

summary(lme.sv)

tab_model(lme.sv)

## Middle Cerebral Artery Velocity

lme.mcav <- lme(MCAV_change ~ LBNP_level + LBNP_level*Treatment,

random = ~ 1|Subject,

na.action = na.omit,

data = df)

summary(lme.mcav)

tab_model(lme.mcav)

## Tolerance to simulated blood loss

df_2 <- df %>%

filter(Period == "Baseline") # Time_to_decompensation is identical for all values of Period.

m <- coxme(Surv(Time_to_decompensation, Status) ~ Treatment + (1 | Subject),

data = df_2)

summary(m)

tab_model(m)
